# Supplementary material for: Specific Gut Microbial Environment in Lard Diet-Induced Prostate Cancer Development and Progression
Source: Int J Mol Sci. 2022 Feb 17;23(4):2214. doi: 10.3390/ijms23042214 (PMC8878430; doi:10.3390/ijms23042214)
Supplement: Supplementary file 1 [file ijms-23-02214-s001.zip › IJMS-1491359 Hiromi Sato Supporting Information PDF.pdf]

Supporting Information

Specific Gut Microbial Environment in Lard Diet-Induced Prostate Cancer Development and Progression

Hiromi Sato [1], Shintaro Narita [1,*], Masanori Ishida [1], Yoshiko Takahashi [1], Huang Mingguo [1], Soki Kashima [1], Ryohei Yamamoto [1], Atsushi Koizumi [1], Taketoshi Nara [1], Kazuyuki Numakura [1], Mitsuru Saito [1], Toshiaki Yoshioka [2] and Tomonori Habuchi [1]

[1]Department of Urology, Akita University School of Medicine, 010-8543, Akita, Japan; hiromisato2002@yahoo.co.jp (H.S.); 3602something@gmail.com (M.I.); yopico.t@gmail.com (Y.T.); huangmg@gipc.akita-u.ac.jp (H.M.); s4005534@gmail.com (S.K.); yama815@med.akita-u.ac.jp (R.Y.); akoizumi@med.akita-u.ac.jp (A.K.); taketonr@gipc.akita-u.ac.jp (T.N.); numakura@doc.med.akita-u.ac.jp (K.N.); urosaito@gmail.com (M.S.); thabuchi@gmail.com (T.H.)
[2]Field of Basic Science, Department of Occupational Therapy, Akita University Graduate School of Health Science, 010-8543, Akita, Japan; yoshiokt@med.akita-u.ac.jp (T.Y.)
*Correspondence: nari6202@gipc.akita-u.ac.jp (S.N.); Tel.: +81-18-884-6154

TABLE OF CONTENTS

Supplementary Figure S1: (A) Total daily calorie intake in the Pten KO mice fed with lard diet or fish oil diet. (B) Total daily calorie intake in the TRAMP-C2 allograft mice fed with lard diet or fish oil diet.

Supplementary Figure S2: Taxonomic composition and relative abundance of microbial groups of each sample at the phylum level. Related to Figure 3A.

Supplementary Figure S3: Taxonomic composition and relative abundance of microbial groups of each sample at the order level. Related to Figure 3B.

Supplementary Figure S4: Abundance histograms of the orders Clostoridiales (A) and Lactobacillales (B). Each bar represents the relative abundance of the specific taxa in an individual mouse.

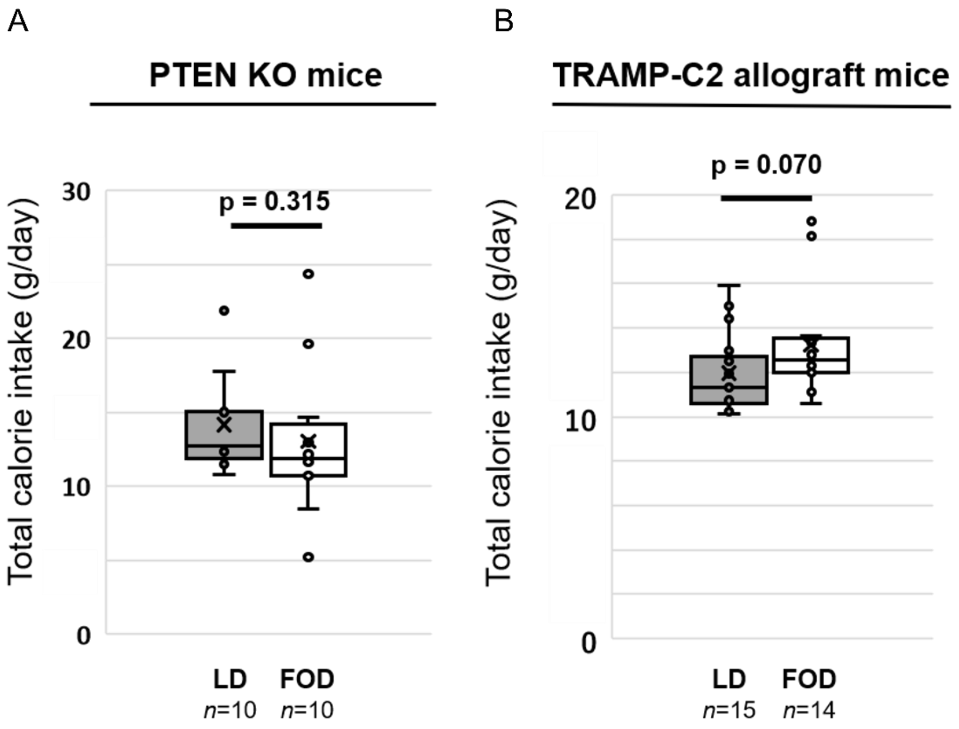

Supplementary Figure S1: (A) Total daily calorie intake in the Pten KO mice fed with lard diet or fish oil diet (n = 10, 10, respectively). (B) Total daily calorie intake in the TRAMP-C2 allograft mice fed with lard diet or fish oil diet (n = 15, 14, respectively).

The line within a box indicates the median value. The round dots and cross marks indicate the value of each sample and the mean value, respectively.

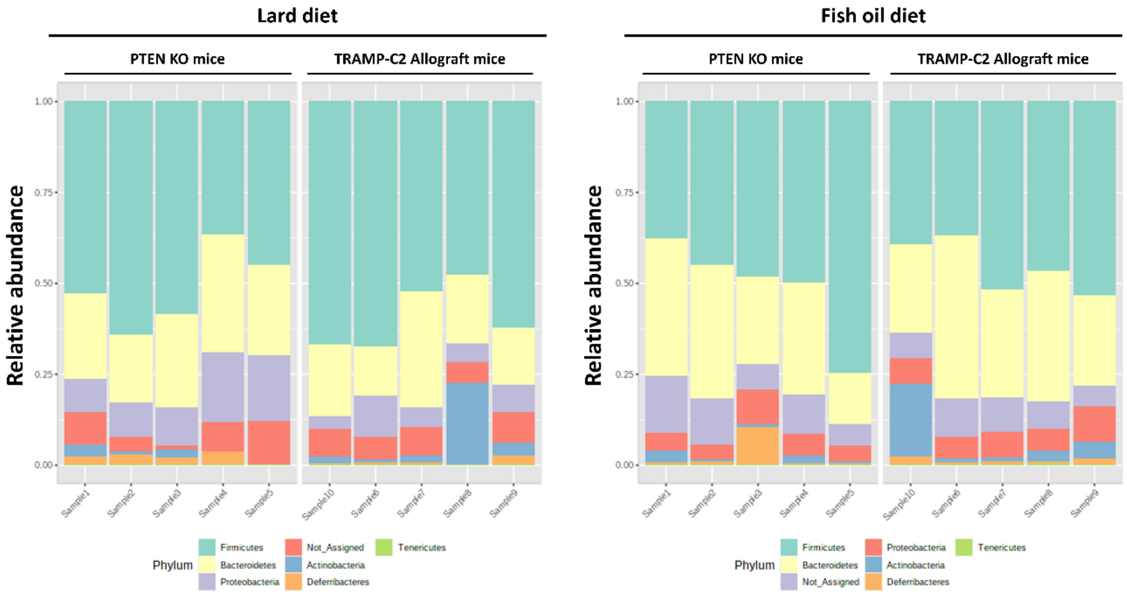

Supplementary Figure S2: Taxonomic composition and relative abundance of microbial groups of each sample at the phylum level. Related to Figure 3A.

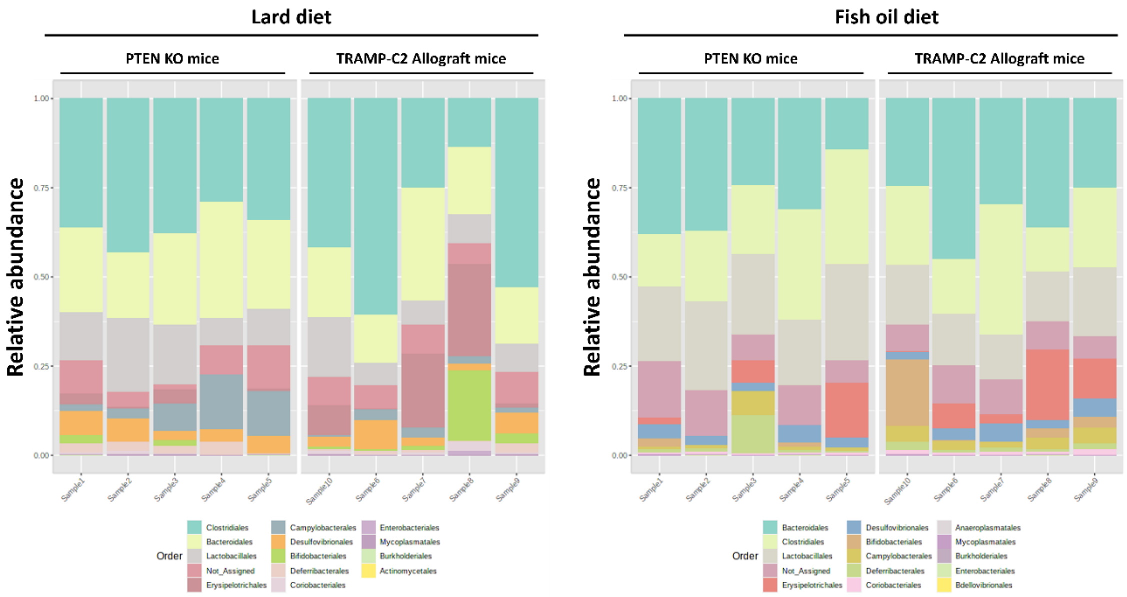

Supplementary Figure S3: Taxonomic composition and relative abundance of microbial groups of each sample at the order level. Related to Figure 3B.

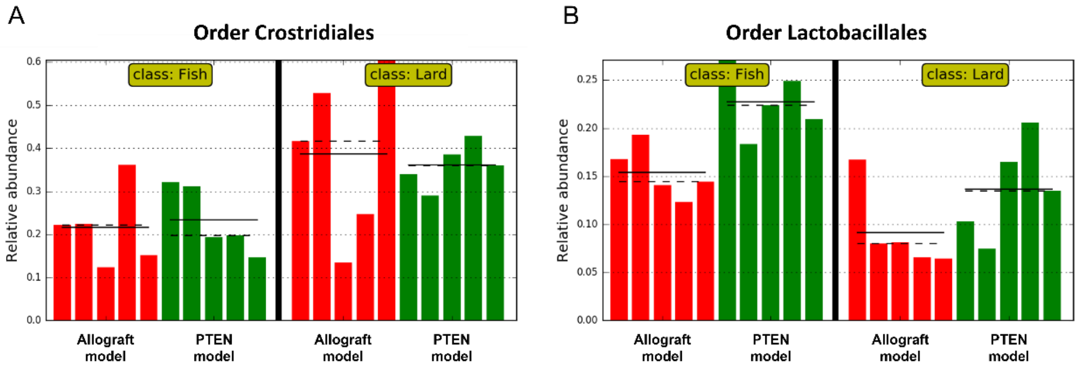

Supplementary Figure S4: Abundance histograms of the orders Clostoridiales (A) and Lactobacillales (B).

Each bar represents the relative abundance of the specific taxa in an individual mouse.
